# Association between cardiometabolic index and frailty among patients with diabetes mellitus: a cross-sectional study

**DOI:** 10.3389/fnut.2024.1495792

**Published:** 2024-12-06

**Authors:** Yi Wei, Jiangyi Yu

**Affiliations:** Department of Endocrinology, Jiangsu Province Hospital of Chinese Medicine, Affiliated Hospital of Nanjing University of Chinese Medicine, Nanjing, China

**Keywords:** diabetes mellitus, frailty, NHANES, cross-sectional study, cardiometabolic index

## Abstract

**Background:**

Cardiometabolic index (CMI) is a novel marker that can assess metabolic status. Studies have found that people with diabetes mellitus (DM) are at high risk of developing frailty. However, there is a lack of evidence between CMI and the risk of frailty in patients with DM. Therefore, the aim of this study was to investigate the association between CMI and frailty in patients with DM.

**Methods:**

This study utilized data from the 2005-2018 National Health and Nutrition Examination Survey (NHANES). Weighted multivariate logistic regression was conducted in this study to explore the association between CMI and frailty status in patients with DM. In addition, subgroup analyses and interaction analyses were conducted to assess heterogeneity between different subgroups. Subsequently, restricted cubic spline (RCS) was also used to test for non-linear relationships.

**Results:**

This study ultimately included 2,761 patients with DM. Weighted multivariate logistic regression showed that, after adjusting all covariates, an increase in the level of CMI was associated with an increased risk of being in a frailty status in patients with DM (OR = 1.12, 95% CI = 1.04–1.22, *p* = 0.005). Dividing CMI into tertiles, the risk of frailty in patients in the highest tertile (Q3) was higher than that of patients in Q1 (OR = 1.56, 95% CI = 1.18–2.07, *p* = 0.002). The non-linear relationship between CMI and the risk of frailty in DM patients was further confirmed by RCS analysis.

**Conclusion:**

This study found that the higher the CMI, the higher the risk of frailty in DM patients. Maintaining a healthy low-fat dietary pattern and properly controlling blood lipid levels may reduce the risk of frailty in patients with DM.

## 1 Introduction

Diabetes mellitus (DM) is a common chronic metabolic disorder, the incidence of which has increased significantly over the past few decades (1, 2). DM is one of the fastest-growing global health problems of the 21st century, imposing a heavy economic burden on society (3, 4). Recently, frailty has attracted much attention in the field of DM (5, 6). Frailty is an emerging public health problem worldwide. The prevalence of frailty in patients with DM is as high as 48%, and the probability of developing frailty is three to five times higher than that in non-DM populations (7). A large-scale meta-analysis conducted by Kong et al. (8) found that the proportions of community-dwelling elderly patients with DM who were in the frail and pre-frail states were 20.1 and 49.1%, respectively (8). Sanz’s team conducted an observational study in several hospitals in Spain to assess malnutrition in elderly patients with DM using the mini nutritional assessment (MNA), and found that the risk of malnutrition reached 39.1% and malnutrition was present in 21.2% of elderly DM patients (9). Also, the team found that malnutrition, albumin and MNA scores were strongly associated with mortality and length of hospitalization. This shows that frailty has become a non-negligible complication of DM. It not only increases the risk of adverse events such as fractures, falls, and hospitalization in patients with DM, but can also substantially increase healthcare expenditure (10). Hanlon et al. (11) suggested that frailty assessment should be incorporated into the routine management of type 2 DM (T2DM) in middle-aged and elderly people.

In 2015, Japanese scholar Wakabayashi proposed the cardiometabolic index (CMI), which is obtained by multiplying the triglycerides (TG)/high-density lipoprotein cholesterol (HDL-C) ratio by waist-to-height ratio (WHtR). CMI can assess the combined effects of body fat distribution and serum lipid levels and is now considered a new method of valuation of visceral adipose tissue (12). CMI has good diagnostic ability for various metabolic disorders including hyperuricaemia (13), liver fibrosis and non-alcoholic fatty liver disease (NAFLD) (14).

Although a non-linear association between CMI and impaired fasting plasma glucose (FPG), insulin resistance and T2DM has been demonstrated (15). However, there is still a lack of information on the association between CMI and the risk of frailty in diabetic patients. CMI is a new type of composite indicator that is convenient, easy to use and cost-effective. Active exploration of the relationship between the two may allow for more effective monitoring of whether diabetic patients are in a frailty state to facilitate effective disease management.

## 2 Materials and methods

### 2.1 Study population and data sources

**Figure 1:**
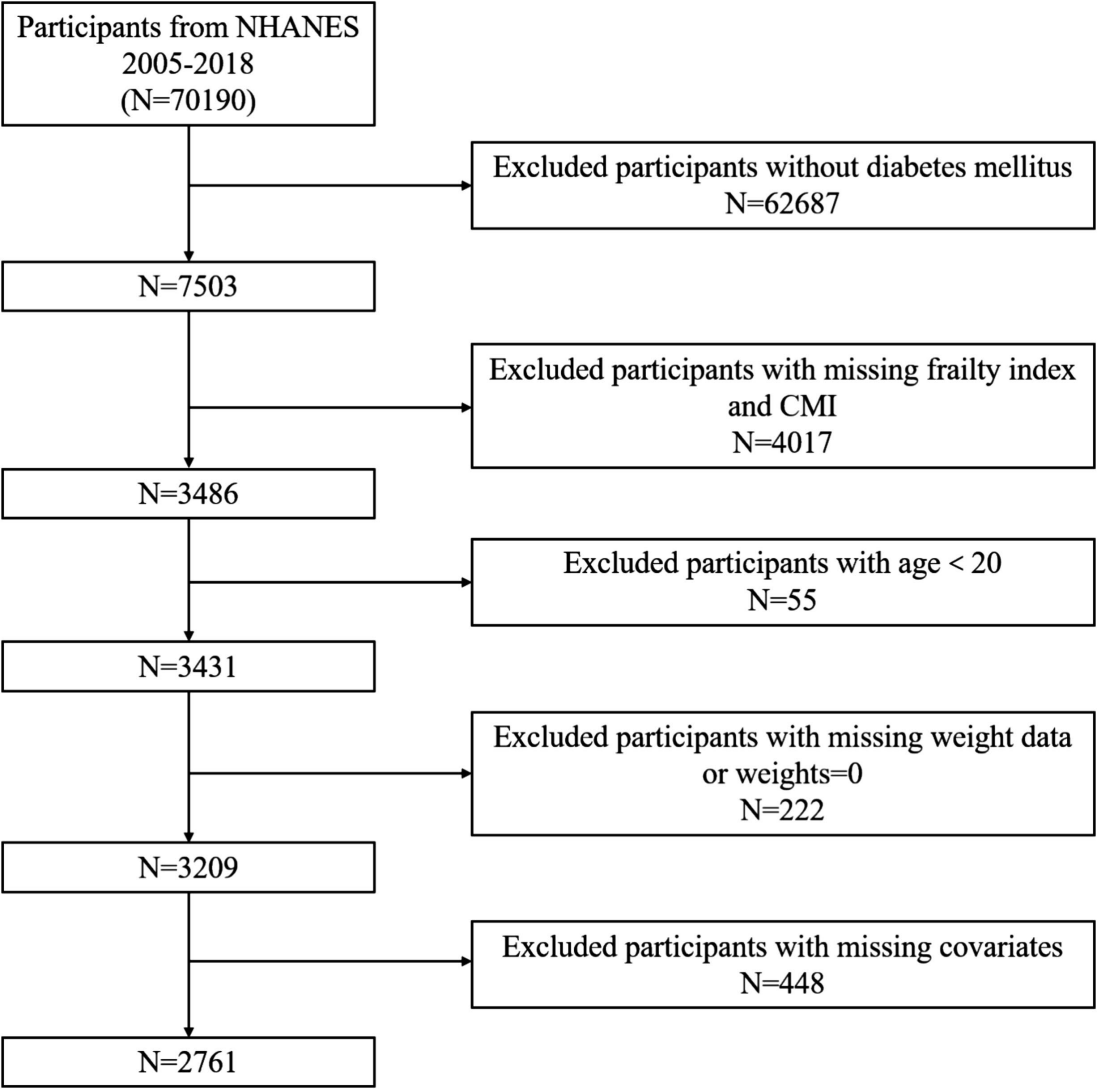
The flowchart of participant selection.

From 2005 to 2018, there were 70,190 participants in the NHANES dataset. Figure 1 illustrates the participant exclusion process with the following criteria: (1) participants who did not meet the diagnostic criteria for DM; (2) age under 20 years; (3) missing complete CMI variables; (4) missing frailty index (FI) variables; (5) participants with missing weight data or weights equal to 0; (6) participants with missing covariates. After excluding these factors, this study ultimately included 2,761 participants who met the criteria.

### 2.2 Diagnostic criteria for DM

Referring to American Diabetes Association Guidelines and previous similar studies, a diagnosis of DM may be made if any of the following conditions are present (16): (1) the individual reports having been diagnosed with DM by a physician; (2) the individual is currently taking glucose-lowering medications or taking insulin injections; (3) a random blood glucose reading of 11.1 mmol/L or higher; (4) a glycated hemoglobin (HbA1c) level of 6.5% or higher; (5) a FPG level of 7.0 mmol/L or higher; or (6) a 2-h blood glucose reading of 11.1 mmol/L or higher on an oral glucose tolerance test (OGTT).

### 2.3 Frailty assessment

The FI was used in order to evaluate the degree of frailty. This approach was originally pioneered by Searle et al. (17). In the present study, we calculate FI following the method constructed by Hakeem et al. (18). The FI is a comprehensive assessment tool covering seven domains and 49 items, including cognition, dependence, depressive symptoms, comorbidities, physical performance and anthropometry, hospital utilization and access to care, and laboratory values. Detailed information can be found in Supplementary Table S1. The severity of each insufficiency was evaluated on a scale ranging from 0 to 1 to determine its level of severity. In this context, 0 denotes the absence of conditions, whereas 1 denotes the most severe. In order to discriminate between debilitating states, we employed a threshold of 0.21. This value refers to a large number of previous studies (19–21). Values that were more than or equal to 0.21 indicated that the individual was experiencing frailty, whereas values that were below this threshold indicated that the individual was not experiencing frailty status.

### 2.4 CMI

Cardiometabolic index (CMI) is a new composite index calculated using a combination of HDL-C, TG, waist circumference (WC) and height. The specific calculation is publicized as follows: CMI = [WC (cm)/height (cm)] × (TG/HDL-C) (12).

### 2.5 Covariates

To investigate the independent association between CMI and risk of frailty in patients with DM, we considered a range of confounding factors that might influence this relationship. Among the demographic and health-related factors were age, gender, race, marital status, education level and family income to poverty ratio (PIR). We classified participants’ race into five categories: Mexican American, Non-Hispanic Black, Non-Hispanic White, other Hispanic, and other race (including multi-racial). Educational level was divided into three categories: below high school, high school, and above high school. Marital status is simply divided into married and other statuses. Smoking status was determined based on self-reported responses. The definitions are as follows: never smoker, former smoker and current smoker. Participants’ total energy intake was obtained through dietary surveys (data from 24-h dietary recall was used in this study). Laboratory test data included serum albumin (Sal).

### 2.6 Statistical analysis

In the NHANES research, each statistical analysis used sample weights, stratification, and clustering as components (22, 23). To do this, weights are chosen, and the official recommendations for the NHANES suggest that the first step is to determine which variable represents the smallest demographic group, and then to choose the weights that correspond to that variable.

Within this particular context, continuous variables are expressed as weighted mean ± standard error (SE), and a *t*-test was used to discriminate between the two groups. For the sake of comparison, categorical variables were rendered in the form of numbers and weighted percentages, and chi-square tests were used. In order to classify the CMI, it was divided into tertiles that ranged from the lowest (Q1) to the highest (Q3). In order to investigate the connection between CMI and frailty status, three different weighted logistic regression models were used. Model 1 did not adjust for any covariates. Model 2 adjusted for age, gender, and race. Model 3 was based on Model 2 and adjusted even further for other covariates, including education level, marital status, PIR, smoke, total energy intake and Sal. A trend test was carried out after CMI was transformed from a continuous variable into a tertile group. The restricted cubic spline (RCS) method was used, and possible confounding factors were taken into consideration in order to investigate the possibility of non-linear relationships between CMI and frailty status in diabetic patients. In addition, we calculated *P* for non-linear. Research was also carried out in the form of subgroup analyses and interaction studies. Statistical significance was determined by using *p* values less than 0.05.

## 3 Results

### 3.1 Baseline characteristics of the study population

Finally, we included 2,761 participants. The mean age of these included participants was 59.06 ± 0.36 years, and 1,440 were male (50.07%). The population was divided according to whether they were in a frailty status or not, and comparisons between groups were made, and the demographic data and laboratory test indices of the participants in each group are shown in Table 1. Notably, there were significant differences in age, gender, race, marital status, education level, BMI, PIR, smoking status, TG, WC, height, WHtR, FPG, total energy intake, Sal, CMI, FI, and the presence of hypertension in DM patients in a frailty status compared to those in a non-frailty status (*p* < 0.05). In the present study, it was found that DM patients in frailty status had higher CMI. They also possessed higher BMI, WC, WHtR and FI, while total energy intake and Sal were significantly lower.

**Table 1:** Baseline characteristics of the study participants (weighted).

Variables	Total (*N* = 2,761)	Non-frailty (*N* = 1,536)	Frailty (*N* = 1,225)	*p*-value
Age, y	59.06(0.36)	57.79(0.51)	60.88(0.45)	<0.001
Gender, *n*(%)				<0.001
Male	1,440(50.07)	897(57.42)	543(39.58)	
Female	1,321(49.93)	639(42.58)	682(60.42)	
Race, *n*(%)				0.019
Mexican American	486(9.07)	291(9.69)	195(8.17)	
Other Hispanic	300(5.69)	173(5.63)	127(5.78)	
Non-Hispanic White	1,109(65.48)	579(65.51)	530(65.44)	
Non-Hispanic Black	617(12.96)	323(11.61)	294(14.90)	
Other Race	249(6.80)	170(7.56)	79(5.72)	
Marital status, *n*(%)				<0.001
Married	1,592(60.51)	959(64.68)	633(54.57)	
Others	1,169(39.49)	577(35.32)	592(45.43)	
Education level, *n*(%)				<0.001
Below high school	885(21.82)	434(18.31)	451(26.82)	
High school	683(27.32)	382(27.14)	301(27.57)	
Above high school	1,193(50.86)	720(54.55)	473(45.61)	
BMI, kg/m^2^	32.78(0.20)	32.06(0.26)	33.82(0.28)	<0.001
PIR	2.77(0.05)	3.04(0.05)	2.38(0.07)	<0.001
Smoke, *n*(%)				0.004
Never	1,395(50.21)	827(53.25)	568(45.87)	
Former	919(33.75)	498(33.32)	421(34.35)	
Current	447(16.05)	211(13.43)	236(19.78)	
TG, mmol/L	1.86(0.05)	1.79(0.05)	1.98(0.08)	0.025
HDL-C, mmol/L	1.27(0.01)	1.27(0.01)	1.27(0.02)	0.757
CMI	1.19(0.04)	1.10(0.04)	1.32(0.07)	0.005
WC, cm	110.70(0.46)	109.32(0.61)	112.66(0.66)	<0.001
Height, cm	167.65(0.30)	168.94(0.40)	165.80(0.37)	<0.001
WHtR	0.661(0.003)	0.648(0.003)	0.680(0.004)	<0.001
FPG, mmol/L	8.219(0.080)	8.216(0.104)	8.224(0.119)	0.963
Sal, g/L	41.41(0.10)	42.04(0.11)	40.51(0.16)	<0.001
Frailty index	0.208(0.003)	0.141(0.002)	0.304(0.003)	<0.001
Total energy intake, kcal	2009.49(24.48)	2059.52(34.93)	1938.17(35.33)	0.02
Hypertension, *n*(%)				<0.001
Yes	1773(63.74)	793(52.22)	980(80.17)	
No	988(36.26)	743(47.78)	245(19.83)	

Abbreviation: BMI, body mass index; PIR, poverty income ratio; TG, triglyceride; CMI, cardiometabolic index; HDL-C, high-density lipoprotein-cholesterol; WC, waist circumference; WHtR, waist-to-height ratio; FPG, fasting plasma glucose; Sal, serum albumin.

In addition, the baseline characteristics of the population according to the tertiles of CMI are demonstrated in Supplementary Table S2. We found that participants in the highest tertile (Q3) had a higher FI compared to the other two groups.

### 3.2 Association between CMI and frailty status

To assess the association between CMI and frailty status in patients with DM, we used three weighted multivariate logistic regression models (Table 2). In the Methods section, we describe in detail the covariates to be adjusted for in each model. All three models found a positive association with frailty status when CMI was used as a continuous variable. For each unit increase in CMI from model 1 to model 3, the risk of DM patients being in a frailty status increased by 9,14, and 12%, respectively.

**Table 2:** The association between CMI and frailty status in patients with diabetes mellitus (weighted).

Exposure	Model 1 OR (95%CI), *P-*value	Model 2 OR (95%CI), *p-*value	Model 3 OR (95%CI), *p-*value
CMI	1.09 (1.02,1.17) 0.01	1.14 (1.05,1.24) 0.001	1.12 (1.04,1.22)0.005
CMI			
Q1	1.00 (ref)	1.00 (ref)	1.00 (ref)
Q2	1.09 (0.85,1.40) 0.50	1.11 (0.86,1.43) 0.44	1.03 (0.79,1.34) 0.83
Q3	1.45 (1.13,1.84) 0.003	1.66 (1.28,2.15) <0.001	1.56 (1.18,2.07) 0.002
*P* for trend	0.002	<0.001	0.001

Model 1: non adjusted.

Model 2: age, gender, race.

Model 3: age, gender, race, education level, marital status, PIR, smoke, total energy intake and Sal.

To ensure the accuracy of our results, we performed sensitivity analyses to examine the relationship between tertile-classified CMI and frailty status. We found that CMI still showed a positive association with frailty status when used as a categorical variable. Also, the risk of frailty status would increase with increasing CMI (*P* for trend < 0.05). In Model 3, diabetic patients in the high CMI group were more likely to be in a frailty status compared to participants in the Q1 CMI groups (OR = 1.56, 95%CI = 1.18–2.07, *p* = 0.002).

In addition, we conducted multivariate logistic regression analyses with FI as a continuous variable and found that CMI was positively correlated with FI in all three models as well (Supplementary Table S3).

**Figure 2:**
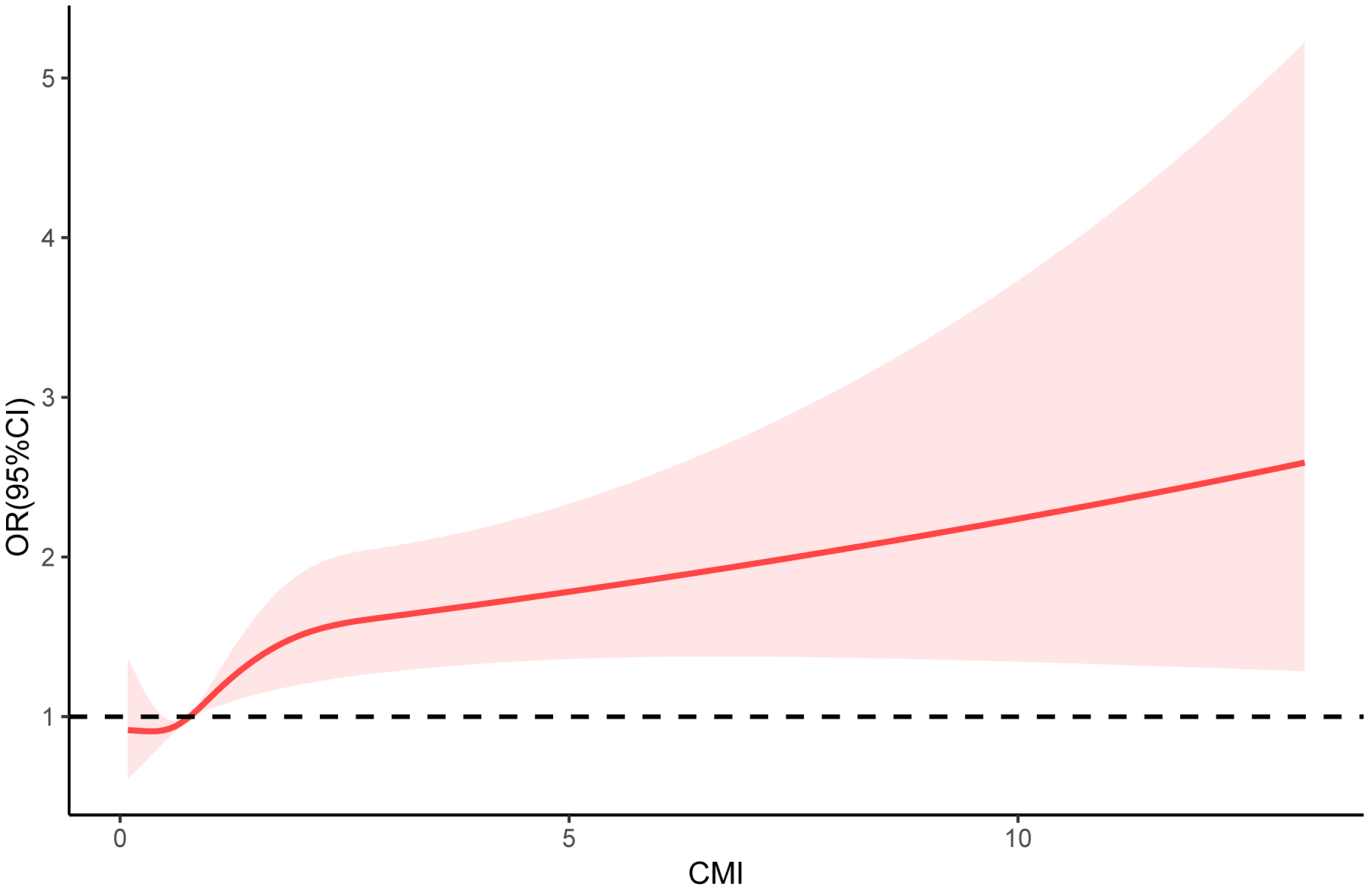
Restricted cubic spline (RCS) regression analyses were used to explore the relationship between CMI and frailty status in patients with DM.

In the RCS analysis, after adjusting for covariates, there was still a significant non-linear relationship between CMI and frailty status (*P* for non-linear = 0.0076) (Figure 2).

### 3.3 Subgroup analysis

In order to determine whether or not there was a correlation between CMI and frailty status in various subgroups of the population, we conducted subgroup analyses and interaction analyses (Table 3). We found that CMI maintained a positive correlation with frailty status in all subgroups. However, no interaction was observed in all subgroups (*P* for interaction > 0.05).

**Table 3:** Subgroup analysis for the association between CMI and frailty status in patients with diabetes mellitus.

	OR 95% CI	*P*-value	*P* for interaction
Age			0.85
<60	1.11(1.02,1.21)	0.02	
≥60	1.10(0.96,1.27)	0.17	
Gender			0.08
Male	1.06(0.97,1.16)	0.21	
Female	1.27(1.04,1.56)	0.02	
Education level			0.57
Below High School	1.11(1.03,1.21)	0.01	
High School	1.17(0.98,1.41)	0.09	
Above High School	1.13(1.00,1.28)	0.05	
Smoke			0.25
Never	1.22(1.05,1.41)	0.01	
Former	1.02(0.91,1.15)	0.69	
Current	1.13(0.95,1.34)	0.16	
PIR level			0.68
<1.3	1.13(0.98,1.31)	0.09	
1.3–3.5	1.11(0.99,1.24)	0.08	
≥3.5	1.14(0.97,1.33)	0.11	

## 4 Discussion

It has been noted that frailty has become the third most common complication in DM patients after macrovascular and microvascular lesions (24). The health burden associated with frailty can no longer be ignored. To our knowledge, this is the first cross-sectional study to explore the association between CMI and the risk of frailty in DM patients. In this cross-sectional study, which included 2,761 patients with DM, there was a non-linear positive association between CMI and frailty status. The findings suggest a trend toward a higher risk of frailty in DM patients as CMI increases. This relationship remained unchanged after adjusting for covariates.

We reviewed and summarized previous studies on the relationship between CMI and DM. Wakabayashi et al. proposed that CMI is a new indicator that can reflect both obesity and lipids and can be used for the identification of DM (12). Their team’s study found that HbA1c was significantly higher in the highest tertile CMI group than in other lower tertiles in both male and female. In addition, a strong correlation between CMI and hyperglycaemia and DM was noted. Song et al. (15) found a non-linear positive correlation between CMI and insulin resistance, impaired fasting glucose, and DM, by analyzing data on adults in the United States. Shi et al. (25) analyzed 11,478 participants from a rural area of northeastern China, and found that an increase in CMI was associated with an increase in the chances of developing DM. CMI could be a useful and economical indicator for screening and quantifying DM in the general Chinese population. Zha et al. (26) found that CMI was positively associated with the risk of DM in the Japanese adult population. They also found that CMI interacted with gender, BMI, exercise habits, and smoking status. Using data from the China Health and Retirement Longitudinal Study (CHARLS), the risk of T2DM was shown to be considerably greater in individuals with a high CMI level in the Chinese population of middle-aged and older people than those with low CMI levels, according to the findings of Qiu et al. (27).

In addition to being a dynamic process, the development of frailty is distinguished by the fact that it may be reversed (28). Early screening of individuals who are at a high risk of developing frailty, in conjunction with early intervention, has the potential to successfully prevent the start of frailty as well as its progression. An extensive number of research teams are now engaged in the process of building risk prediction models in order to evaluate the likelihood of frailty developing in diabetic patients. By incorporating characteristics such as marital status, WC, cognitive capacity, grip strength, and social activities, Bu et al. constructed a nomogram model to predict the degree of frailty in patients with DM (29). This model was able to accurately predict the degree of frailty in these patients. There is a substantial correlation between frailty and the prognosis of diabetic patients, since frailty is a prevalent occurrence in diabetic patients. He et al. analyzed data from CHARLS and the English Longitudinal Study of Aging (ELSA) and found that frailty is associated with the development of DM in pre-DM and increases the risk of cardiovascular disease and all-cause mortality in patients with DM in the pre-DM stage and in those with DM (30). Early detection of frailty and timely implementation of targeted interventions may therefore be effective in reducing the burden associated with DM (31).

Notably, CMI is a convenient and feasible marker in clinical practice to help early screening of patients at risk of frailty. CMI is a comprehensive measure of obesity-related disease that integrates indicators of abdominal obesity and dyslipidemia, both of which are key drivers of metabolic disorders. Abdelhafiz and Sinclair (32) found that compared to non-frail patients with DM, those in the frail state tended to have a higher body weight, WC, and BMI, and to be less physically active, have elevated cholesterol elevated cholesterol levels, and also have more comorbidities. An exploratory trial conducted by Simpson et al. (33) found that the rate of aging in DM patients could be slowed and FI levels lowered through weight control. Weight loss through nutritional interventions, physical activity and even the use of medications (e.g., metformin) can improve or even reverse the debilitating state of DM patients (34). On the other hand, abnormal accumulation of lipids leads to the development of an inflammatory response (35). Numerous previous studies have shown that chronic low-grade inflammation is strongly associated with the development of DM and frailty. There is a direct link between frailty and elevated levels of inflammation, marked by elevated C-reactive protein (CRP) and interleukin (IL)-6 (36, 37). Therefore, lipid monitoring and weight management in patients with DM is helpful in reducing the risk of developing frailty.

There are several strengths of our study. To begin, it is the first study to investigate the connection between CMI levels and frailty status in patients with DM. This study offers fresh perspectives on the connection between metabolic features and nutritional status. Second, using the NHANES data, we strictly followed a complex sampling design for weighted analyses to ensure that the results are representative of the wider population. This also greatly increases the generalizability and applicability of the present results. However, in order to reduce the influence of any confounding factors on the findings of the studies, the current research included adjustments for potential variables. This allowed the researchers to shed light on the independent association that exists between CMI and frailty. Because of this, the findings of the present research have significant repercussions for public health policies that aim to recognize frailty status at an early stage and prevent it from occurring in a timely manner.

We should also acknowledge certain limitations of this study. Firstly, the sample for the research was restricted to a particular demographic region, which may restrict the extent to which the results may be generalized to other communities. Therefore, in order to increase the scope of the article’s generalizable application, data from more sources need to be added for analysis in future studies. Secondly, the present study is a cross-sectional study and it is not possible to examine whether the relationship between CMI and frailty is causal. Therefore, the results derived from this study still need to be validated by large-scale prospective studies. Furthermore, while adjusting for a number of covariates, there are still potential common factors that have not been taken into account. Overall, while our study provides valuable insights, these limitations should be taken into account when interpreting the results.

## 5 Conclusion

Frailty was much more likely to occur in DM patients who had higher levels of CMI. CMI can be used as a practical tool to identify DM patients who are at risk of frailty. This indicator emphasizes the importance of proper control of lipid levels and body weight in DM patients, and helps guide patients with DM to adhere to a healthy low-fat dietary pattern. At the same time, CMI could help in early screening for frailty risk, thus enabling timely interventions to slow disease progression. Future large-scale prospective studies are needed to validate the findings of this study.

## Data Availability

Publicly available datasets were analyzed in this study. This data can be found at: NHANES (https://wwwn.cdc.gov/nchs/nhanes/default.aspx).
