# Supplementary material for: Association between cardiometabolic index and frailty among patients with diabetes mellitus: a cross-sectional study
Source: Front Nutr. 2024 Dec 6;11:1495792. doi: 10.3389/fnut.2024.1495792 (PMC11660792; doi:10.3389/fnut.2024.1495792)
Supplement: Supplementary file 1 [file Table_1.docx]

**Supplementary Tables**

Table S1. The 49-Item Defects to Calculate Frailty Index

| **Defects** | **Score** |
| --- | --- |
| **Cognition** |  |
| 1. Experience confusion/memory problems | Yes=1；No=0 |
| **Dependence** |  |
| 2. Managing money | Difficulty=1  No Difficulty=0 |
| 3. Stooping, crouching, kneeling |  |
| 4. Lifting or carrying |  |
| 5. House chore |  |
| 6. Preparing meals |  |
| 7. Standing up from armless chair |  |
| 8. Getting in and out of bed difficulty |  |
| 9. Using fork, knife, drinking from cup |  |
| 10. Dressing yourself |  |
| 11. Standing for long periods difficulty |  |
| 12. Grasp/holding small objects |  |
| 13. Attending social event |  |
| 14. Push or pull large objects |  |
| 15. Walking for a quarter mile difficulty |  |
| 16. Walking up 10 steps difficulty |  |
| **Depressive Symptoms** |  |
| 17. Have little interest in doing things | Nearly every day = 1,  More than half the days = 0.66,  Several days = 0.33,  Not at all = 0 |
| 18. Feeling down, depressed, or hopeless |  |
| 19. Trouble sleeping or sleeping too much |  |
| 20. Feeling tired or having little energy |  |
| 21. Poor appetite or overeating |  |
| 22. Feeling bad about yourself |  |
| 23. Trouble concentrating on things |  |
| **Comorbidities** |  |
| 24. Arthritis | No = 0,  Suspect = 0.5,  Yes = 1 |
| 25. Thyroid problems |  |
| 26. Chronic bronchitis |  |
| 27. Cancer |  |
| 28. Congestive heart failure |  |
| 29. Coronary heart disease |  |
| 30. Angina |  |
| 31. Heart attack |  |
| 32. Stroke |  |
| 33. Blood pressure |  |
| 34. Diabetes |  |
| 35. Weak/failing kidneys |  |
| 36. Urinary Leakage |  |
| **Hospital Utilization and Access to Care** |  |
| 37. Self-rated health | Fair or poor = 1; excellent, very good, or good = 0 |
| 38. Health now compared with 1 year ago | Worse = 1; About the same, better = 0 |
| 39. Overnight hospital patient in past year | Yes=1；No=0 |
| 40. Frequency of health care use during past year | None = 0, 1-5 = 0.5, >5 = 1 |
| 41. Number of prescribed medications | None = 0, 1-4 = 0.5, ≥5 = 1 |
| **Physical Performance and Anthropometry** |  |
| 42. Body mass index | <18.5 or ≥30 = 1, 25 to 30 = 0.5, 18.5 to 25 = 0 |
| 43. Handgrip strength | Male:  For BMI≤24, GS≤29 = 1;  For BMI 24.1 to 28, GS≤30 = 1;  For BMI >28, GS≤32 = 1.  Female:  For BMI≤23, GS≤17 = 1;  For BMI 23.1 to 26, GS≤17.3 = 1;  For BMI 26.1 to 29, GS≤18 = 1;  For BMI>29, GS≤21 = 1. |
| **Laboratory Values** |  |
| 44. Glycohemoglobin (%) | 0% to 5.7% = 0, >5.7% = 1 |
| 45. Red blood cell count (million cells/ml) | Male: 4.7 to 6.1 = 0, Other = 1  Female: 4.2 to 5.4 = 0, Other = 1 |
| 46. Hemoglobin (g/dL) | Male: 13.5 to 18 = 0, Other = 1  Female: 12 to 16 = 0, Other = 1 |
| 47. Red cell distribution width (%) | 11.6 to 14.6 = 0, Other = 1 |
| 48. Lymphocyte percent (%) | 20 to 40 = 0, Other = 1 |
| 49. Segmented neutrophils percent (%) | 40 to 80 = 0, Other = 1 |

Notes: BMI = Body mass index; GS = grip strength.

**Table S2:** Baseline characteristics of study participants according to tertile groups of CMI (weighted)

| **Variables** | **Total**  **(*N* =2761)** | **Q1** | **Q2** | **Q3** | ***P*-value** |
| --- | --- | --- | --- | --- | --- |
| **Age, y** | 59.06(0.36) | 60.35(0.56) | 60.77(0.63) | 56.61(0.57) | < 0.0001 |
| **Gender, n(%)** |  |  |  |  | 0.284 |
| Male | 1440(50.07) | 464(47.73) | 459(49.14) | 517(52.74) |  |
| Female | 1321(49.93) | 456(52.27) | 462(50.86) | 403(47.26) |  |
| **Race, n(%)** |  |  |  |  | < 0.0001 |
| Mexican American | 486(9.07) | 111(6.21) | 166(9.37) | 209(11.15) |  |
| Other Hispanic | 300(5.69) | 82(4.99) | 112(6.74) | 106(5.40) |  |
| Non-Hispanic White | 1109(65.48) | 318(60.75) | 348(62.53) | 443(71.77) |  |
| Non-Hispanic Black | 617(12.96) | 320(21.34) | 203(13.69) | 94 5.53) |  |
| Other Race | 249(6.80) | 89(6.71) | 92(7.67) | 68(6.15) |  |
| **Marriage status, n(%)** |  |  |  |  | 0.208 |
| Married | 1592(60.51) | 510(57.00) | 537(62.23) | 545(61.95) |  |
| Others | 1169(39.49) | 410(43.00) | 384(37.77) | 375(38.05) |  |
| **Education level, n(%)** |  |  |  |  | 0.315 |
| Below high school | 885(21.82) | 266(19.11) | 304(24.49) | 315(21.82) |  |
| High school | 683(27.32) | 230(27.71) | 226(26.08) | 227(28.02) |  |
| Above high school | 1193(50.86) | 424(53.18) | 391(49.43) | 378(50.16) |  |
| **BMI, kg/m^2^** | 32.78(0.20) | 29.60(0.33) | 33.10(0.30) | 35.13(0.34) | < 0.0001 |
| **PIR** | 2.77(0.05) | 2.90(0.08) | 2.67(0.09) | 2.75(0.08) | 0.096 |
| **Smoke, n(%)** |  |  |  |  | 0.026 |
| Never | 1395(50.21) | 514(55.06) | 479(51.74) | 402(44.97) |  |
| Former | 919(33.75) | 271(29.91) | 311(33.72) | 337(36.90) |  |
| Current | 447(16.05) | 135(15.03) | 131(14.53) | 181(18.13) |  |
| **Sal, g/L** | 41.41(0.10) | 41.69(0.17) | 41.33(0.15) | 41.25(0.16) | 0.127 |
| **TG, mmol/L** | 1.86(0.05) | 0.90(0.01) | 1.47(0.02) | 2.98(0.09) | < 0.0001 |
| **HDL-C, mmol/L** | 1.27(0.01) | 1.59(0.03) | 1.26(0.01) | 1.02(0.01) | < 0.0001 |
| **CMI** | 1.19(0.04) | 0.36(0.00) | 0.78(0.01) | 2.20(0.08) | < 0.0001 |
| **WC, cm** | 110.70(0.46) | 102.20(0.75) | 111.58(0.69) | 116.90(0.77) | < 0.0001 |
| **Height, cm** | 167.65(0.30) | 167.50(0.47) | 167.21(0.48) | 168.14(0.50) | 0.342 |
| **WHtR** | 0.661(0.003) | 0.611(0.004) | 0.668(0.004) | 0.696(0.004) | < 0.0001 |
| **FPG, mmol/L** | 8.22(0.08) | 7.54(0.10) | 7.99(0.11) | 8.96(0.16) | < 0.0001 |
| **Total energy intake, kcal** | 2009.49(24.48) | 1963.17(37.84) | 1918.61(35.14) | 2122.23(49.34) | 0.003 |
| **Frailty index** | 0.208(0.003) | 0.197(0.004) | 0.206(0.005) | 0.219(0.004) | < 0.001 |
| **Frailty Status** |  |  |  |  | 0.008 |
| No | 1536(58.77) | 547(62.79) | 524(60.76) | 465(53.85) |  |
| Yes | 1225(41.23) | 373(37.21) | 397(39.24) | 455(46.15) |  |
| **Hypertension, n(%)** |  |  |  |  | 0.221 |
| Yes | 1773(63.74) | 588(60.76) | 580(63.72) | 605(66.20) |  |
| No | 988(36.26) | 332(39.24) | 341(36.28) | 315(33.80) |  |

Abbreviation: BMI, body mass index; PIR, poverty income ratio; TG, triglyceride; CMI, cardiometabolic index; HDL-C, high-density lipoprotein-cholesterol; WC, waist circumference; WHtR, waist-to-height ratio; FPG, fasting plasma glucose; Sal, serum albumin

**Table S3:** The association between CMI and frailty index in patients with diabetes mellitus (weighted)

| Exposure | Model I  β (95%CI) *P*-value | Model II  β (95%CI) *P*-value | | Model III  β (95%CI) *P*-value | |
| --- | --- | --- | --- | --- | --- |
| CMI | 0.004(0.002,0.006) <0.001 | | 0.006(0.003,0.008) <0.001 | | 0.005(0.003, 0.007) <0.001 |
| CMI |  | |  | |  |
| Q1 | ref | | ref | | ref |
| Q2 | 0.009(-0.003,0.020) 0.139 | | 0.009(-0.002,0.020) 0.110 | | 0.004(-0.007, 0.014) 0.481 |
| Q3 | 0.022(0.011,0.033) <0.001 | | 0.029(0.018,0.040) <0.001 | | 0.023(0.013, 0.033) <0.001 |
| *P* for trend | <0.001 | | <0.001 | | <0.001 |

Model I: Non adjusted

Model II: Age, Gender, Race

Model III: Age, Gender, Race, Education level, Marital status, PIR, Smoke, total energy intake and Sal
